# Supplementary figures and images for: The seroincidence of childhood Shigella sonnei infection in Ho Chi Minh City, Vietnam
Source: PLoS Negl Trop Dis. 2023 Oct 30;17(10):e0011728. doi: 10.1371/journal.pntd.0011728 (PMC10635567; doi:10.1371/journal.pntd.0011728)

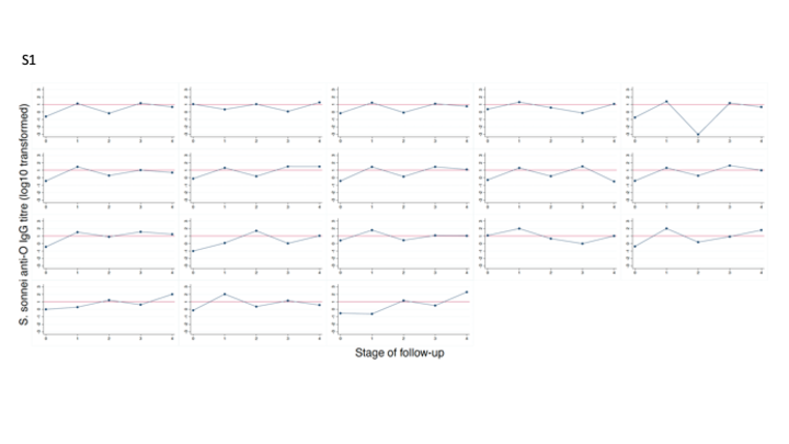

Supplement: S1 Fig — Scatter plots showing the variation in S. sonnei anti-O IgG titres by stage of follow-up for the 18 participants with completed follow-up that had evidence of >1 seroconversion event during two years of observation. (TIF) [file pntd.0011728.s001.tif]

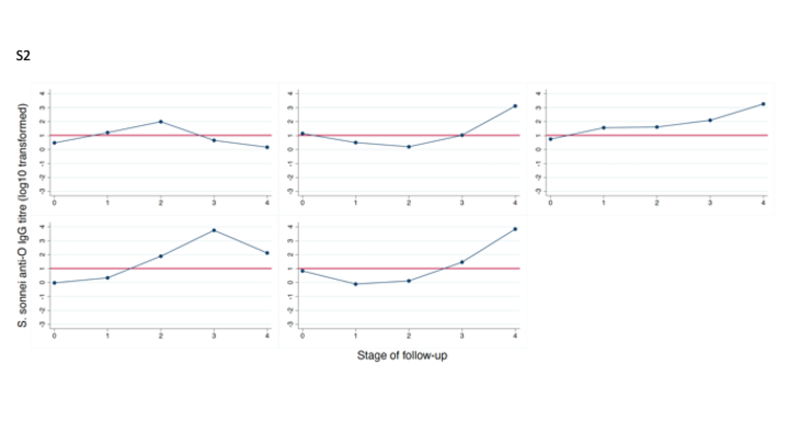

Supplement: S2 Fig — Scatter plots showing the variation in S. sonnei anti-O IgG titres by stage of follow-up for the five participants with completed follow-up that had evidence of consecutive four-fold rises in S. sonnei anti-O IgG after a presumed single seroconversion event. (TIF) [file pntd.0011728.s002.tif]

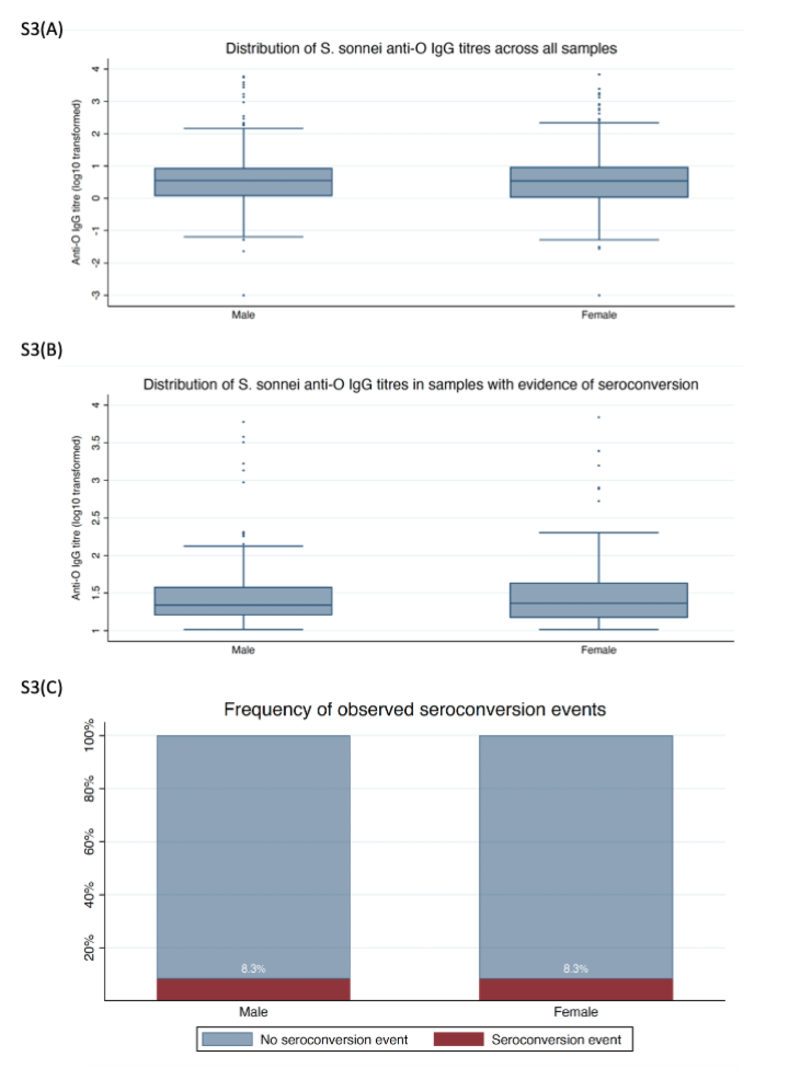

Supplement: S3 Fig — Distributions of S. sonnei anti-O IgG titres and the freqeuncy of observed seroconversion in participants of different sexes. A, All S. sonnei anti-O IgG titres (log10-transformed EU), inclusive of seronegative samples and those from participants with incomplete follow-up. B, S. sonnei anti-O IgG titres (log10-transformed EU) in convalescent samples with evidence of seroconversion only, inclusive of samples taken from participants with incomplete follow-up. C, Frequency of observed seroconversion events in convalescent samples, inclusive of samples taken from participants with incomplete follow-up. (TIF) [file pntd.0011728.s003.tif]

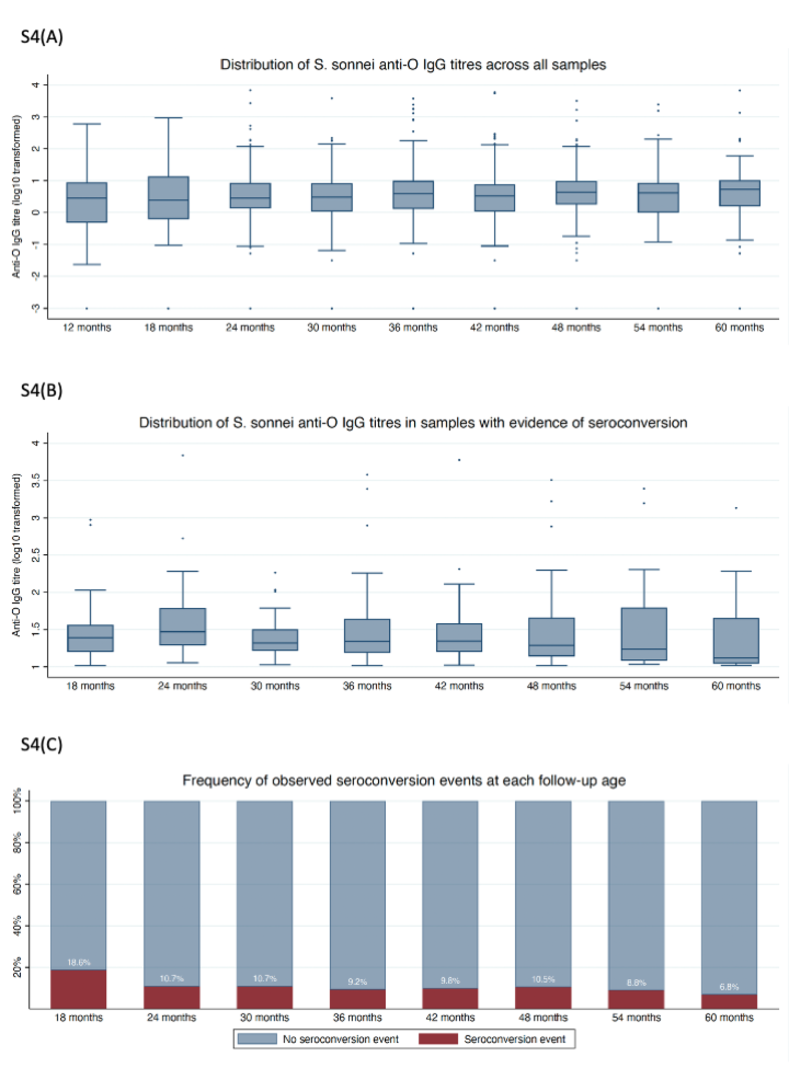

Supplement: S4 Fig — Distributions of S. sonnei anti-O IgG titres and the freqeuncy of observed seroconversion in participants of different ages at the time of sampling. A, All S. sonnei anti-O IgG titres (log10-transformed EU), inclusive of seronegative samples and those from participants with incomplete follow-up. B, S. sonnei anti-O IgG titres (log10-transformed EU) in convalescent samples with evidence of seroconversion only, inclusive of samples taken from participants with incomplete follow-up. C, Frequency of observed seroconversion events in convalescent samples, inclusive of samples taken from participants with incomplete follow-up. (TIF) [file pntd.0011728.s004.tif]

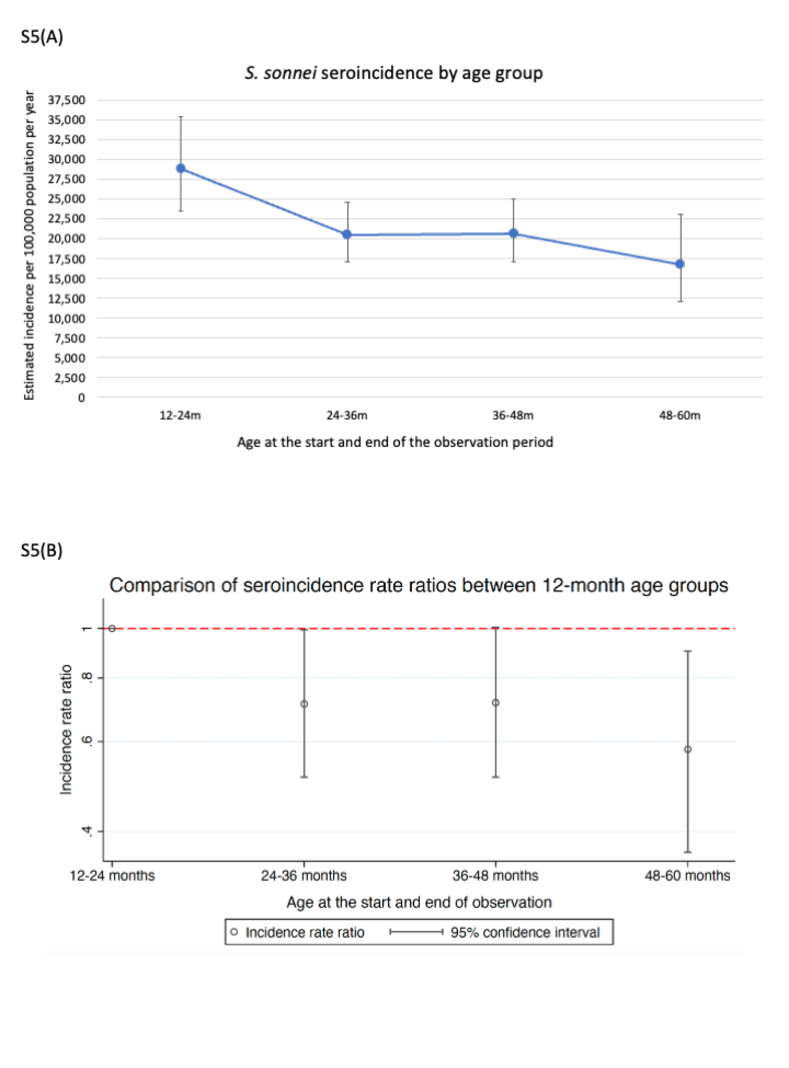

Supplement: S5 Fig — S. sonnei seroincidence in different 12-month age groups, shown as: A, Exposures per 100,000 population, with 95% confidence intervals. B, Seroincidence rate ratios, with the 12–124 month age period as a reference category. (TIF) [file pntd.0011728.s005.tif]
