# Supplementary material for: The seroincidence of childhood Shigella sonnei infection in Ho Chi Minh City, Vietnam
Source: PLoS Negl Trop Dis. 2023 Oct 30;17(10):e0011728. doi: 10.1371/journal.pntd.0011728 (PMC10635567; doi:10.1371/journal.pntd.0011728)
Supplement: S1 Text — Table showing the baseline characteristics of participants that did not attend all follow-up visits. Table B. Frequency distribution of seroconversion events and variation in median anti-O IgG levels by sex. Table showing the distribution of observed seroconversion events and median anti-O IgG levels in participants of different sexes. Table C. Frequency distribution of seroconversion events and variation in median anti-O IgG levels by age at the time of sampling. Table showing the distribution of observed seroconversion events and median anti-O IgG levels in participants of different ages at the time of conalescent serum sampling. Table D. Frequency distribution of seroconversion events and variation in median anti-O IgG levels by calendar month of sampling. Table showing the distribution of observed seroconversion events and median anti-O IgG levels in different calander months of convalescent serum sampling. Table E. Factors associated with anti-O IgG measurements in all samples. Table showing unadjusted and adjusted regression coefficients for anti-O IgG titres across all serum samples. Multivariable adjustment was made for sex, age at the time of serum sampling, calander month of serum sampling and calandar month of ELISA testing. Table F. Factors associated with anti-O IgG measurements in convalescent samples at the point of observed seroconversion. Table showing unadjusted and adjusted regression coefficients for anti-O IgG titres across all convalescent serum samples in which seroconversion was deemed to have occurred (i.e. a newly positive result). Multivariable adjustment was made for sex, age at the time of serum sampling, calander month of serum sampling and calandar month of ELISA testing. Table G. Numbers of participants, seroconversion events and total observation times used in the seroincidence calculations for 6-month age groups. Table showing the cumulative number of participants, number of person years of observation, and number of seroconversion [file pntd.0011728.s006.docx]

**Table A. Baseline characteristics of participants with incomplete follow-up**

|  |  | Participants with incomplete follow-up  (n=114) |
| --- | --- | --- |
| Sex | Male | 62 (54.4%) |
|  | Female | 52 (45.6%) |
| Age at enrolment (months) | 12 | 39 (34.2% |
|  | 18 | 14 (12.3%) |
|  | 24 | 16 (14.0% |
|  | 30 | 23 (20.2%) |
|  | 36 | 22 (19.3%) |
| Calendar month of baseline sampling | January | 0 (0.0%) |
|  | February | 6 (5.3%) |
|  | March | 1 (0.9%) |
|  | April | 1 (0.9%) |
|  | May | 1 (0.9%) |
|  | June | 9 (7.9%) |
|  | July | 30 (26.3%) |
|  | August | 15 (13.2%) |
|  | September | 15 (13.2%) |
|  | October | 12 (10.5%) |
|  | November | 13 (11.4%) |
|  | December | 11 (9.7%) |
| Obesity | Yes | 6 (5.3%) |
|  | No | 71 (62.2%) |
|  | Missing | 37 (32.5%) |
| Malnourishment | Yes | 2 (1.7%) |
|  | No | 75 (65.8%) |
|  | Missing | 37 (32.5%) |
| History of past or present breast feeding | Yes | 105 (92.1%) |
|  | No | 9 (7.9%) |
| Active breast feeding at enrolment | Yes | 13 (11.4%) |
|  | No | 101 (88.6%) |
| Age at breastfeeding cessation (months) | Median | 5.0 |
|  | IQR | 2.0 – 8.0 |
| Time since breastfeeding cessation (months) | Median | 17.0 |
|  | IQR | 9.0 – 25.0 |
| Age of first solid food (months) | Median | 6.0 |
|  | IQR | 5.0 – 6.0 |
| Time since first solid food (months) | Median | 18.0 |
|  | IQR | 6.5 – 24.0 |
| Weight (Kg) | Median | 11.2 |
|  | IQR | 10.0 – 13.6 |
| Height (cm) | Mean | 84.5 |
|  | SD | 76.0 – 90.0 |
| History of prior hospital admissions | Yes | 16 (14.0%) |
|  | No | 98 (86.0%) |
| Number of prior hospital admissions (if admitted) | Median | 1.0 |
|  | IQR | 1.0 – 1.0 |
| Self-reported prior episode of dysentery | Yes | 4 (3.5%) |
|  | No | 110 (96.5%) |
| Prior diagnosis of being underweight or stunted | Yes | 7 (6.1%) |
|  | No | 106 (93.0)%) |
|  | Missing | 1 (0.9%) |
| Regular use of probiotics | Yes | 44 (38.6%) |
|  | No | 70 (61.4%) |
| Number of adults (age ≥15 years) in household | Median | 4.0 |
|  | IQR | 3.0 – 6.0 |
| Number of children (age <15 years) in household | Median | 2.0 |
|  | IQR | 1.0 – 2.0 |
| Grandparents present in household | Yes | 79 (69.3%) |
|  | No | 35 (30.7%) |
| Attendance at school or kindergarten | Neither | 83 (72.8%) |
|  | School | 25 (21.9%) |
|  | Kindergarten | 6 (5.3%) |
| School or kindergarten hours per week, if an attendee | Median | 48.0 |
|  | IQR | 40.0 – 48.0 |
| Average monthly household income (Vietnamese dong) | <1 million | 0 (0%) |
|  | 1-3 million | 6 (5.3%) |
|  | 3-5 million | 25 (21.9%) |
|  | 5-10 million | 56 (49.1%) |
|  | >10 million | 27 (23.7%) |
| Main household source of drinking water | Piped to residence | 68 (59.7%) |
|  | Piped to public tap | 0 (0.0%) |
|  | Bottled water | 44 (38.5%) |
|  | Well in residence | 2 (1.8%) |
|  | Public well | 0 (0.0%) |
|  | Rain water | 0 (0.0%) |
|  | Spring | 0 (0.0%) |
|  | River/Stream | 0 (0.0%) |
|  | Other | 0 (0.0%) |
| Household water source for washing vegetables | Piped to residence | 106 (93.0%) |
|  | Piped to public tap | 0 (0.0%) |
|  | Bottled water | 1 (0.9%) |
|  | Well in residence | 5 (4.4%) |
|  | Public well | 2 (1.7%) |
|  | Rain water | 0 (0.0%) |
|  | Spring | 0 (0.0%) |
|  | River/Stream | 0 (0.0%) |
|  | Other | 0 (0.0%) |
| Variation in water source between wet and dry season | Yes | 2 (1.7%) |
|  | No | 112 (98.3%) |
| Tendency for household water shortages | Yes | 5 (4.4%) |
|  | No | 109 (95.6%) |
| Water storage on the household premises | None | 46 (40.3%) |
|  | Inside storage | 22 (19.4%) |
|  | Outside storage | 46 (40.3%) |
|  | Inside and outside storage | 0 (0.0%) |
| Coverage of water stored at the household premises | Yes | 67 (58.8%) |
|  | No | 1 (0.9%) |
|  | N/A or missing | 46 (40.3%) |
| Main method of water retrieval from household water store | Pouring or tap | 55 (48.3%) |
|  | Scooping cup with short handle | 7 (6.1%) |
|  | scooping cup with long handle | 5 (4.4%) |
|  | Other | 1 (0.9%) |
|  | N/A or missing | 46 (40.3%) |
| Use of toilet paper in the household | Yes | 62 (54.4%) |
|  | No | 52 (45.6%) |
| Use of toilet spray-wash in the household | Yes | 111 (97.4%) |
|  | No | 3 (2.6%) |
| Soap for handwashing present in the toilet room | Yes | 113 (99.1%) |
|  | No | 1 (0.9%) |
| Tendency for flooding in close proximity to residence | None | 84 (73.7%) |
|  | Seldom | 17 (14.9%) |
|  | Moderate | 12 (10.5%) |
|  | Severe | 1 (0.9%) |
| Residence in close proximity to animals | Yes | 76 (66.7%) |
|  | No | 38 (33.3%) |
| Type of animal in close contact | Chicken | 8 (7.0%) |
|  | Duck | 1 (0.9%) |
|  | Dog | 61 (53.5%) |
|  | Cat | 29 (25.4%) |
|  | Other | 4 (3.5%) |

**Table B. Frequency distribution of seroconversion events and variation in median anti-O IgG levels by sex**

| Sex | Results of convalescent samples | | Median (IQR) anti-O IgG level (log10 EU) | |
| --- | --- | --- | --- | --- |
|  | No seroconversion | Seroconversion | All serum samples (including baseline) | Convalescent samples with evidence of seroconversion |
| Male | 1318/1473 (89.5%) | 155/1473 (10.5%) | 3.55 (1.16 - 8.72) | 21.89 (15.99 - 38.30) |
| Female | 1156/1291 (89.5%) | 135/1291 (10.5%) | 3.45 (1.05 - 9.38) | 23.21 (14.84 - 43.50) |
| **Total** | 2474/2764 (89.5%) | 290/2764 (10.5%) | 3.50 (1.12 - 9.15) | 22.18 (15.39 - 40.55) |
|  |  | Pearson’s chi-squared test: *p*=0.955 | K-sample equality-of-medians test: *p*=0.684 | K-sample equality-of-medians test: *p*=0.814 |

**Table C. Frequency distribution of seroconversion events and variation in median anti-O IgG levels by age at the time of sampling**

| Age at the time of serum sampling | Results of convalescent samples | | Median (IQR) anti-O IgG level (log10 EU) | |
| --- | --- | --- | --- | --- |
|  | No seroconversion | Seroconversion | All serum samples (including baseline) | Convalescent samples with evidence of seroconversion |
| 12 months | N/A | N/A | 2.82 (0.48 - 8.80) | N/A |
| 18 months | 184/226 (81.4%) | 42/226 (18.6%) | 2.43 (0.63 - 13.73) | 24.68 (15.93 - 36.42) |
| 24 months | 250/280 (89.3%) | 30/280 (10.7%) | 2.83 (1.37 - 8.48) | 29.66 (19.56 - 61.48) |
| 30 months | 377/422 (89.3%) | 45/422 (10.7%) | 3.03 (1.09 - 8.32) | 20.97 (16.35 - 31.79) |
| 36 months | 511/563 (52%) | 52/563 (9.2%) | 3.91 (1.30 - 9.90) | 21.97 (15.58 - 44.36) |
| 42 months | 424/470 (90.2%) | 46/470 (9.8%) | 3.31 (1.08 - 7.59) | 22.18 (15.99 - 38.30) |
| 48 months | 367/410 (89.5%) | 43/410 (10.5%) | 4.37 (1.79 - 9.70) | 19.40 (13.87 - 45.81) |
| 54 months | 238/261 (91.2%) | 23/261 (8.8%) | 4.17 (1.01 - 8.41) | 17.22 (12.18 - 62.20) |
| 60 months | 123/132 (93.2%) | 9/132 (6.8%) | 5.33 (1.58 - 10.13) | 13.18 (11.13 - 45.12) |
| **Total** | 2474/2764 (89.5%) | 290/2764 (10.5%) | 3.50 (1.12 - 9.15) | 22.18 (15.39 - 40.55) |
|  |  | Pearson’s chi-squared test: *p*=0.006 | K-sample equality-of-medians test: *p*<0.001 | K-sample equality-of-medians test: *p*=0.359 |

**Table D. Frequency distribution of seroconversion events and variation in median anti-O IgG levels by calendar month of sampling**

| Calendar month at the time of serum sampling | Results of convalescent samples | | Median (IQR) anti-O IgG level (log10 EU) | |
| --- | --- | --- | --- | --- |
|  | No seroconversion | Seroconversion | All serum samples (including baseline) | Convalescent samples with evidence of seroconversion |
| January | 179/212 (84.4%) | 33/212 (15.6%) | 5.25 (1.20 - 12.10) | 24.46 (16.49 - 33.46) |
| February | 153/206 (74.3%) | 53/206 (25.7%) | 5.25 (0.74 - 16.19) | 17.49 (14.33 - 29.03) |
| March | 289/322 (89.8%) | 33/322 (10.2%) | 2.62 (0.59 - 9.14) | 18.24 (12.09 - 10.66) |
| April | 236/249 (94.8%) | 13/249 (5.2%) | 1.81 (0.55 - 4.03) | 44.21 (28.18 - 73.04) |
| May | 167/173 (96.5%) | 6/173 (3.5%) | 4.16 (2.27 - 6.84) | 36.71 (24.39 - 54.87) |
| June | 202/218 (92.7%) | 16/218 (7.3%) | 4.74 (2.74 - 7.90) | 120.13 (48.60 - 187.96) |
| July | 170/183 (92.9%) | 13/183 (7.1%) | 1.88 (0.70 - 8.62) | 27.14 (14.12 - 62.20) |
| August | 248/258 (96.1%) | 10/258 (3.9%) | 1.08 (0.28 - 4.55) | 29.20 (12.14 - 201.67) |
| September | 227/246 (92.3%) | 19/246 (7.7%) | 3.56 (1.59 - 9.47) | 19.56 (12.19 - 45.81) |
| October | 228/253 (90.1%) | 25/253 (9.9%) | 4.21 (2.08 - 8.53) | 14.95 (11.44 - 28.65) |
| November | 200/226 (88.5%) | 26/226 (11.5%) | 5.82 (1.63 - 16.35) | 21.67 (15.95 - 26.30) |
| December | 175/218 (80.3%) | 43/218 (19.7%) | 3.83 (1.80 - 12.69) | 24.98 (18.47 - 35.99) |
| **Total** | 2474/2764 (89.5%) | 290/2764 (10.5%) | 3.50 (1.12 - 9.15) | 22.18 (15.39 - 40.55) |
|  |  | Pearson’s chi-squared test: *p*<0.001 | K-sample equality-of-medians test: *p*<0.001 | K-sample equality-of-medians test: *p*<0.001 |

**Table E. Factors associated with anti-O IgG measurements in all samples**

| Variable | Unadjusted | | Adjusted* | |
| --- | --- | --- | --- | --- |
|  | Coefficient | P-value | Coefficient | P-value |
| Sex | 3.03 (-14.33 - 20.39) | 0.73 | 2.55 (-14.80 - 19.91) | 0.77 |
| Age | 0.57 (-0.12 - 1.26) | 0.11 | 0.59 (-0.10 - 1.29) | 0.09 |
| Calendar month of sampling | 2.83 (0.23 - 5.43) | 0.03 | 2.91 (0.27 - 5.55) | 0.03 |
| Calendar month of ELISA | -4.67 (-26.74 - 17.40) | 0.68 | 0.82 (-21.63 - 23.28) | 0.94 |

*Adjusted for all other variables in the table

**Table F. Factors associated with anti-O IgG measurements in convalescent samples at the point of observed seroconversion**

| Variable | Unadjusted | | Adjusted* | |
| --- | --- | --- | --- | --- |
|  | Coefficient | P-value | Coefficient | P-value |
| Sex | 8.01 (-144.39 - 160.41) | 0.92 | 8.84 (-143.86 - 161.54) | 0.91 |
| Age | 2.18 (-4.29 - 8.64) | 0.51 | 1.82 (-4.71 - 8.35) | 0.58 |
| Calendar month of sampling | 13.85 (-4.70 - 32.41) | 0.14 | 14.34 (-4.48 - 33.17) | 0.14 |
| Calendar month of ELISA | -77.11 (-237.58 - 83.35) | 0.07 | -91.99 (-253.74 -69.77) | 0.26 |

*Adjusted for all other variables in the table

**Table G. Numbers of participants, seroconversion events and total observation times used in the seroincidence calculations for 6-month age groups**

| **Age range during period of observation** | | **Number of participants (with completed follow-up)** | | **Duration of follow-up as part of this cohort (person years)** | | **Number of participants with evidence of seroconversion** | | **Number of seroconversion events observed** | | **Estimated seroincidence (infections per 100,000 per year)**  (95% CI) |
| --- | --- | --- | --- | --- | --- | --- | --- | --- | --- | --- |
| **Age cohort** | **Age at enrolment** | **By enrolment age** | **Overall for cohort** | **By enrolment age** | **Overall for cohort** | **By enrolment age** | **Overall for cohort** | **By enrolment age** | **Overall for cohort** |  |
| 12-18m | 12m | 198 | 198 | 99 | 99 | 37 | 37 | 37 | 37 | **37,374**  (28,962 - 48,229) |
| 18-24m | 12m | 198 | 246 | 99 | 123 | 17 | 27 | 17 | 27 | **21,951**  (15,730 - 30,632) |
|  | 18m | 48 |  | 24 |  | 10 |  | 10 |  |  |
| 24-30m | 12m | 198 | 388 | 99 | 194 | 18 | 41 | 18 | 41 | **21,134**  (16,104 - 27,736) |
|  | 18m | 48 |  | 24 |  | 2 |  | 2 |  |  |
|  | 24m | 142 |  | 71 |  | 21 |  | 21 |  |  |
| 30-36m | 12m | 198 | 509 | 99 | 254.5 | 13 | 51 | 13 | 51 | **20,039**  (15,678 - 25,613) |
|  | 18m | 48 |  | 24 |  | 6 |  | 6 |  |  |
|  | 24m | 142 |  | 71 |  | 18 |  | 18 |  |  |
|  | 30m | 121 |  | 60.5 |  | 14 |  | 14 |  |  |
| 36-42m | 18m | 48 | 436 | 24 | 218 | 3 | 44 | 3 | 44 | **20,183**  (15,501 - 26,281) |
|  | 24m | 142 |  | 71 |  | 18 |  | 18 |  |  |
|  | 30m | 121 |  | 60.5 |  | 11 |  | 11 |  |  |
|  | 36m | 125 |  | 62.5 |  | 12 |  | 12 |  |  |
| 42-48m | 24m | 142 | 388 | 71 | 194 | 11 | 41 | 11 | 41 | **21,134**  (16,104 - 27,736) |
|  | 30m | 121 |  | 60.5 |  | 19 |  | 19 |  |  |
|  | 36m | 125 |  | 62.5 |  | 11 |  | 11 |  |  |
| 48-54m | 30m | 121 | 246 | 60.5 | 123 | 12 | 23 | 12 | 23 | **18,699**  (12,936 - 27,031) |
|  | 36m | 125 |  | 62.5 |  | 11 |  | 11 |  |  |
| 54-60m | 36m | 125 | 125 | 62.5 | 62.5 | 8 | 8 | 8 | 8 | **12,800**  (6,702 - 24,448) |
| 12-60m | | 634 | | 1268 | | 254 | | 272 | | **21,451**  (19,307 - 23,834) |

**Table H. Numbers of participants, seroconversion events and total observation times used in the seroincidence calculations for 12-month age groups**

| **Age range during period of observation** | | **Number of participants (with completed follow-up)** | | **Duration of follow-up as part of this cohort (person years)** | | **Number of participants with evidence of seroconversion** | | **Number of seroconversion events observed** | | **Estimated seroincidence in infections per 100,000 per year**  (95% CI) |
| --- | --- | --- | --- | --- | --- | --- | --- | --- | --- | --- |
| **Age cohort** | **Age at enrolment** | **By enrolment age** | **Overall for cohort** | **By enrolment age** | **Overall for cohort** | **By enrolment age** | **Overall for cohort** | **By enrolment age** | **Overall for cohort** |  |
| 12-24m | 12m | 198 | 246 | 198 (1y) | 222 | 54 | 64 | 54 | 64 | **28,829**  (23,446 - 35,448) |
|  | 18m | 48 |  | 24 (0.5y) |  | 10 |  | 10 |  |  |
| 24-36m | 12m | 198 | 509 | 198 (1y) | 448.5 | 31 | 92 | 31 | 92 | **20,513**  (17,096 - 24,612) |
|  | 18m | 48 |  | 48 (1y) |  | 8 |  | 8 |  |  |
|  | 24m | 142 |  | 142 (1y) |  | 39 |  | 39 |  |  |
|  | 30m | 121 |  | 60.5 (0.5y) |  | 14 |  | 14 |  |  |
| 36-48m | 18m | 48 | 436 | 24 (0.5y) | 412 | 3 | 85 | 3 | 85 | **20,631**  (17,071 - 24,933) |
|  | 24m | 142 |  | 142 (1y) |  | 29 |  | 29 |  |  |
|  | 30m | 121 |  | 121 (1y) |  | 30 |  | 30 |  |  |
|  | 36m | 125 |  | 125 (1y) |  | 23 |  | 23 |  |  |
| 48-60m | 30m | 121 | 246 | 60.5 (0.5y) | 185.5 | 12 | 31 | 12 | 31 | **16,712**  (12,120 - 23,043) |
|  | 36m | 125 |  | 125 (1y) |  | 19 |  | 19 |  |  |
| 12-60m | | 634 | | 1268 | | 254 | | 272 | | **21,451**  (19,307 - 23,834) |
