# Supplementary material for: The seroincidence of childhood Shigella sonnei infection in Ho Chi Minh City, Vietnam
Source: PLoS Negl Trop Dis. 2023 Oct 30;17(10):e0011728. doi: 10.1371/journal.pntd.0011728 (PMC10635567; doi:10.1371/journal.pntd.0011728)
Supplement: Attachment — Submitted filename: Responses to reviewer comments.docx [file pntd.0011728.s008.docx]

Dear Editors,

We thank the reviewers for their reviews, which we believe will help to enhance the manuscript.

Please find a detailed point-by-point response to the reviewer comments below.

We are grateful for your continued consideration for publication of this manuscript and look forward to your response.

Yours sincerely,

Dr Nick K Jones

Dr Stephen Baker

**Responses to reviewer comments**

***Methods***

*-Are the objectives of the study clearly articulated with a clear testable hypothesis stated? Is the study design appropriate to address the stated objectives? Is the population clearly described and appropriate for the hypothesis being tested? Is the sample size sufficient to ensure adequate power to address the hypothesis being tested? Were correct statistical analysis used to support conclusions? Are there concerns about ethical or regulatory requirements being met?*

*Reviewer #1:*

*(No Response)*

*Reviewer #2:*

*The objectives of the study are based on logical and testable hypothesis/rationale of the study.*

*The study is scientifically designed to achieve targeted objectives.*

*The characteristics (demographic and clinical features) of study participants are properly described.*

*The present study recruited substantial study subjects (3,498 samples were taken from 748 participants) which makes results more authentic and scientifically sound.*

*Data analysis has been done using standard statistical tools.*

*Ethical approval of study was taken from relevant ethical bodies (Oxford tropical research ethics committee (OxTREC) in the United Kingdom (approval 1058-13) and ethics committee of the Hospital for Tropical Diseases and Hung Vuong Hospital in Ho Chi Minh City,Vietnam).*

Thank you for this summary, which we are in agreement with.

*Reviewer #3:*

*Line 138 - Need to clarify what is meant by "sanitation" in the questionnaire.*

*Line 112 - Need to add further detail about how the study will contribute to the objective of "informing the progression of S. sonnei vaccines" - currently the link is not very clear.*

Line 138 – the term ‘sanitation’ was used to categorise questions relating to toilet spray-wash or paper use and handwashing after using the toilet. We have now re-phrased this as ‘personal hygiene practices’ for clarity (line 141 of the revised manuscript). The baseline data derived from the questionnaire are clearly presented in table 1 of the manuscript for readers to view.

Line 112 – Currently, we rely on bacterial culture as a measure of S. sonnei disease incidence. However, culture is only widely performed in research settings in LMIC and seldom in the diagnosis of routine diarrheal disease. The type of serology used here, which is infrequently exploited for infections with enteric bacteria, provides a mechanism of assessing exposure to S. sonnei and the associated natural antibody history. Given that a S. sonnei vaccine is likely to be dependent on responses to O-antigen, these data can therefore inform vaccine formulation. Additionally, this approach becomes a method to detect exposure, antibody duration and impact of vaccination in future surveillance work. We have amended lines 112-116 to read as follows, for better clarity:

“Here, hypothesizing substantial S. sonnei exposure via asymptomatic infection, and aiming to inform the progression of S. sonnei (or polyvalent Shigella) vaccines by providing insight into circulating levels of naturally occurring anti-O IgG, we sought to measure the seroincidence of S. sonnei in a longitudinal cohort of 748 children aged <5 years residing in Ho Chi Minh City, Vietnam”

--------------------

***Results***

*Does the analysis presented match the analysis plan? Are the results clearly and completely presented? Are the figures (Tables, Images) of sufficient quality for clarity?*

*Reviewer #1: (No Response)*

*Reviewer #2: Results are properly and scientifically presented and analysed as per the experimental strategies/methods. Good quality figures are given in the article containing information which is easily understandable and readable.*

We thank the reviewer for this assessment.

*Reviewer #3: - The results should specify the characteristics of censored participants - i.e. those who didn't complete follow-up*

We have now included this information as a supplementary table (Table S1) and have referenced this in the text on lines 194-195.

--------------------

***Conclusions***

*Are the conclusions supported by the data presented? Are the limitations of analysis clearly described? Do the authors discuss how these data can be helpful to advance our understanding of the topic under study? Is public health relevance addressed?*

*Reviewer #1: (No Response)*

*Reviewer #2:*

*The conclusion supports the data presented.*

*The limitations of the study are absent, the author should include this section in the revised article.*

*Significant finding has been discussed and compared with previous data from literature, so it will further enhance our knowledge of the topic.*

Thank you for this assessment. Although some study limitations had previously been outlined in the discussion section, we have now rearranged the manuscript to have an entire paragraph designated to the discussion of study limitations for greater clarity (lines 303-318 of the revised manuscript).

*Reviewer #3:*

*The discussion briefly mentions limitations in the statistical power of this study (line 273) - this point needs to be further elaborated, particularly as it somewhat contradicts line 291 which states that the study's strength is its large sample size.*

We have now re-worded our description of this limitation on lines 303-305 of the revised manuscript for better clarity:

“although the study’s relatively large sample size provides confidence in the seroincidence estimates reported, the ability to ascertain risk factors for S. sonnei seroconversion was likely limited by inadequate statistical power for identifying associations with small effect sizes.”

*Were clinical characteristic recorded for seroconverters? It would be good to quantify how the sero results compare with the detectable incidence of S. sonnei based on clinical presentations.*

This has now been added to the limitations. We have little data on true diseases incidence as defined by the presence of clinical symptoms. What we describe here is exclusively the seroincidence, with two key observations/questions arising from our findings. The first is that there is an enormous amount of exposure and likely asymptomatic infection. The second is to ask what this means for individuals and whether it is associated with subsequent protective immunity against symptomatic disease. These questions will require a different study design and repeated, longitudinal stool sampling to answer.

*Noting that the IgG response drops to two-fold higher than baseline by 10 weeks, and the threshold used for seroconversion in the study is a four-fold increase, the 6 monthly intervals for sampling reduces the validity of the results and limits the ability of the study to meet its stated objective of measuring exposure to S. sonnei. I recognise that this is noted in the discussion as a limitation, however further information on the level of under-ascertianment is needed to increase the validity of the results.*

Our study design prioritised longevity of follow-up and number of participants enrolled over the frequency of observation points included, in order to provide a unique, representative insight into the minimum longitudinal exposure of infants to S. sonnei. Greater frequency of serum sampling was discussed at the study design stage, but was deemed unethical by the research ethics committee. Importantly, although the rate of under-ascertainment of S. sonnei exposure in the study remains unknown, the estimated S. sonnei seroincidence described is substantial and is highly unlikely to be an over-estimate. We have added more clarity on this limitation in lines 303-312 of the revised manuscript:
“This study has limitations. Firstly, although the study’s relatively large sample size provides confidence in the seroincidence estimates reported, the ability to ascertain risk factors for S. sonnei seroconversion was likely limited by inadequate statistical power for identifying associations with small effect sizes. Similarly, although the longevity of participant follow-up serves as an important study strength in enabling substantial longitudinal surveillance, the necessarily large sampling intervals make it likely for some seroconversion events to have been missed. It has been previously demonstrated that anti-O IgG responses to natural S. sonnei exposure peak at 2 weeks post-infection and drop to roughly two-fold higher than baseline by 10 weeks (25). Nevertheless, this study provides an important estimate of the minimum extent of S. sonnei exposure in this cohort, and although the exact rate of under-ascertainment is unknown, it can be expected to have been evenly distributed across age groups.”

--------------------

***Editorial and Data Presentation Modifications***

*Use this section for editorial suggestions as well as relatively minor modifications of existing data that would enhance clarity. If the only modifications needed are minor and/or editorial, you may wish to recommend “Minor Revision” or “Accept”.*

*Reviewer #1:*

*(No Response)*

*Reviewer #2:*

*(No Response)*

*Reviewer #3:*

*In figures 1A-1C, remove the word "simple" in the y-axis title.*

The y-axis of these figures reads ‘sample count’ (not ‘simple count’), alluding to the total number of samples collected.

--------------------

***Summary and General Comments***

*Use this section to provide overall comments, discuss strengths/weaknesses of the study, novelty, significance, general execution and scholarship. You may also include additional comments for the author, including concerns about dual publication, research ethics, or publication ethics. If requesting major revision, please articulate the new experiments that are needed.*

*Reviewer #1:*

*S. flexneri and S. sonnei have been identified as the predominant species of Shigella worldwide. S. sonnei specifically dominates in developed areas and is the second most common species after S. flexneri in economically disadvantaged populations. The proportion of S. sonnei has been progressively increasing over the years.*

*In this study, the ELISA method was employed, utilizing IgG antibodies against the S. sonnei O-antigen, to estimate the incidence of childhood exposure to S. sonnei in Ho Chi Minh City, where S. sonnei was the prevailing Shigella species. This study provided intriguing insights, and the information it provided holds significant importance.*

*However, it is well-known that serotyping methods for bacteria in the Enterobacteriaceae family, including Shigella, often encounter issues with cross-reactivity, leading to false-positive results. Therefore, to validate the conclusions drawn from the serological approach employed in this study, it is recommended to incorporate additional methods such as fluorescent quantitative PCR or bacterial isolation. These approaches can help address potential methodological errors and compare the incidence rate of Shigella infections and the rate of exposure to Shigella among populations.*

Many thanks for these comments, but we respectfully disagree. The points made do not take into account the study question, design or observations.

1. We aimed to investigate the seroepidemiology of S. sonnei O-antigen in this population, which we know is likely to be substantially greater than disease incidence.
2. We know from previous work that S. sonnei O-antigen is highly specific for S. sonnei, exhibiting cross-reactivity only with Plesiomonas shigelloides (Shepherd et al. *Infect Immun*. 2000). The antigen used was detoxified O-antigen, which has been shown to exhibit limited cross-reactivity with other enteric bacteria. The cross-reactivity with Plesiomonas is known, but Plesiomonas is contracted exclusively from contaminated water, while S. sonnei is transmitted person-to-person. Given the sanitation conditions and water quality in Ho Chi Minh City, we know Plesiomonas exposure to be implausible and therefore conclude that the antibody responses measured are specific to repeating sugars of the O-antigen. We have added a sentence to our methods to clarify this justification for selecting anti-O as our serological marker of interest (lines 149-151): “Anti-O IgG was selected because of a known specificity to detect S. sonnei seroconversion and limited cross reactivity with other species of enterobacteriaceae, aside from the waterborne organism, Plesiomonas shigelloides (15)”
3. Lastly, this was a longitudinal study, aiming to measure antibody natural history and is unlikely to correlate with symptomatic disease or faecal carriage. PCR on stool was considered pointless, given potential exposure and carriage of a range of enteric pathogens including Shigella and E. coli. We agree that this would be of general interest, but it was outside of the scope of this study’s design.

*Reviewer #2:*

*In the current article (PNTD-D-23-00760), the authors highlighted the high sero-incidence of S. sonnei in Ho Chi Minh City, Vietnam. The theme and information of this article will be of great interest for readers and scientific community to know cases of Shigellosis due to S. sonnei and thus will be helpful for regional public health authorities to make treatment and prevention strategies.*

*Though the study is scientifically designed and executed, but the following comments should be properly addressed.*

*(i)Inclusion and exclusion criteria for selection of participant may be added.*

The cohort of infants enrolled in the study was sought from an existing cohort that had been assembled in 2009 for a study of viral infections in Vietnamese infants and children. The inclusion and exclusion criteria for that study are described here: Anders KL, Nguyen NM, Van Thuy NT, Hieu NT, Nguyen HL, Hong Tham NT, et al. A birth cohort study of viral infections in Vietnamese infants and children: study design, methods and characteristics of the cohort. BMC Public Health. 2013 Oct 8;13(1):937.

We have added this reference in our methods section (line 129 of the revised manuscript). Due to the antenatal nature of recruitment, exclusion criteria were based on maternal characteristics only; girls aged <15 years, those intending to relocate within 12 months of enrolment, and women known to be HIV positive. No additional exclusion criteria were applied at the point of enrolment into the present study, and we have clarified this point on line 130 of the revised manuscript.

*(ii) The limitation of the study should be added in the article.*

Although some study limitations had previously been outlined in the discussion section, we have now rearranged the manuscript to have an entire paragraph designated to the discussion of study limitations for greater clarity (lines 303-318 of the revised manuscript).

*(iii) The conclusion should be revised focusing on the main findings of the study and their clinical applications including future prospects.*

Thank you for this suggestion. We have now amended the concluding paragraph to highlight important implications for future Shigella vaccine implementation (lines 324-344 of the revised manuscript).

*Reviewer #3:*

*This is a good paper and easy to read. However the 6-month sampling interval is not conducive to meeting the stated objectives of measuring sero-incidence given that IgG levels drop to two-fold above baseline within 2.5 months, and the threshold for seroconversion used in this study is a four-fold increase.*

Thank you for this observation. We have now detailed more clearly in the limitations paragraph of the discussion section that potential under-ascertainment is an important caveat to interpreting our results (lines 306-312). Importantly, our seroincidence estimates serve as valid estimates of the *minimum* S. sonnei seroincidence in the observed population and demonstrate substantial levels of pathogen exposure.
